# Measurement of subcutaneous fat tissue: reliability and comparison of caliper and ultrasound via systematic body mapping

**DOI:** 10.1038/s41598-022-19937-4

**Published:** 2022-09-22

**Authors:** Jana Hoffmann, Jens Thiele, Stefan Kwast, Michael Andrew Borger, Thomas Schröter, Roberto Falz, Martin Busse

**Affiliations:** 1 Institute of Sports Medicine and Prevention, University of Leipzig, 04103 Leipzig, Germany; 2 Department of Radiology, Helios Klinik, 04435 Schkeuditz, Germany; 3 University Department of Cardiac Surgery, Heart Center Leipzig, 04289 Leipzig, Germany

**Keywords:** Medical research, Risk factors, Anatomy, Disease prevention, Fat metabolism

## Abstract

Caliper and ultrasound (US) are used to measure subcutaneous fat tissue depth (SFT) and then to calculate total body fat. There is no evidence-based recommendation as to whether caliper or US are equally accurate. The aim of this paper was therefore to compare reliability of both methods. In this methodical study, 54 participants (BMI: 24.8 ± 3.5 kg/m^2^; Age: 43.2 ± 21.7 years) were included. Using systematic body mapping, the SFT of 56 areas was measured. We also analyzed 4 body sites via MRI. A comparison between caliper and US detected clear differences in mean SFT of all areas (0.83 ± 0.33 cm vs. 1.14 ± 0.54 cm; p < 0.001) showing moderate reliability (ICC 0.669, 95%CI: 0.625–0.712). US and MRI revealed in the abdominal area a SFT twice as thick as caliper (2.43 ± 1.36 cm vs. 2.26 ± 1.32 cm vs. 1.15 ± 0.66 cm; respectively). Caliper and US revealed excellent intrarater (ICC caliper: 0.944, 95%CI: 0.926–0.963; US: 0.934, 95%CI: 0.924–0.944) and good interrater reliability (ICC caliper: 0.794, 95%CI: 0.754–0.835; US: 0.825, 95%CI: 0.794–0.857). Despite the high reliability in measuring SFT that caliper and US show, our comparison of the two methods yielded clear differences in SFT, particularly in the abdominal area. In accuracy terms, US is preferable for most mapping areas.

## Introduction

Body composition is highly relevant when assessing health and nutritional condition^1^. Especially in professional sports as well as in the medical context, the fat mass represents a decisive factor for the evaluation of body weight and its compartments^2^. Adipose tissue consists of subcutaneous adipose tissue (SAT) and visceral adipose tissue (VAT), the latter rather of medical and clinical relevance due to its metabolic characteristics^3^. Total body fat (TBF), on the other hand, plays an essential role to assess sports and nutrition intervention effects^4^. This is particularly important when the gain or loss of body fat determines the success of training and therapy. In this context, BMI is not suitable for classifying body weight, since no statement can be derived about muscle or fat mass. For future clinical application, the difference between TBF and SAT may indicate VAT. There are various methods to measure fat tissue, such as MRI, DXA, bioimpedance or caliper, all of which depend on the objective as well as the device’s accuracy and availability. CT and MRI are used to quantify SAT and VAT by multiplying the volume by the slice thickness based on certain gray-scale image segmentation. The MRI is considered as gold standard for body fat content and its distribution^5^. DXA on the other hand is approved as an ionising method to determine body fat^6^. These methods are characterized by very high accuracy, but are not available for daily use due to their expense and poor cost-to-clinical-benefit ratio^7^. Hence, there are several other ways to quantify body fat more easily.

Calipometry is an easy and well-tested method that measures the subcutaneous fat tissue depth (SFT) through skinfold thickness to determine total body fat applying a specific skinfold equation, which calculates TBF on the basis of body density manifested through regression analysis^8^. Additionally, the ultrasound (US) device can be used to differentiate subcutaneous from visceral fat, and has been applied for skinfold measurements as well^9^. Störchle et al.^10^ reported that US yields the most accurate SAT measurements thanks to essentially higher image resolution than MRI (0.1 mm vs. 1.3 mm).

However, historically, the correlations between TBF and skinfolds were usually arrived at after caliper use^8^. Consequently, we need clarification as to whether ultrasound (US) and caliper measurements of subcutaneous fat are equally accurate.

Although several studies have already been carried out on this topic, their results are inconsistent, which is not surprising considering the innumerable skinfold equations and varying statistical methods applied^11–13^. Contradictory results indicate an interaction between the number of skinfolds, body type, participants’ age and applied method. In contrast to previous approaches, clarification is needed as to whether if measurement devices are interchangeable.

This is the first study to measure skinfolds via systematic body mapping by US and caliper.

The aims of the study were: (a) Intrarater and interrater reliability of US and caliper, (b) SFT comparison between both methods (and in 4 cases with MRI) of various body types. We expected a difference between methods depending on the thickness of subcutaneous fat.

## Materials and methods

### Ethical aspects

This study was conducted in accordance with the latest revision of the Declaration of Helsinki and was approved by the Ethics Committee of the Medical Faculty, University of Leipzig (097/17-ek). All participants received an information letter and signed written informed consent forms. Two experienced sport scientists carried out the examinations.

### Participants

This study included 54 participants aged 43.2 ± 21.7 years with a BMI between 17 and 32 kg/m^2^ (Table 1). To ensure a heterogeneous study population, we aimed to enroll participants with various body types.

**Table 1:** Anthropometric data.

	Men (n = 26)	Women (n = 28)	Total sample (n = 54)	
	x̅ ± SD	x̅ ± SD	x̅ ± SD	Range (min–max)
Age (years)	42.8 ± 22.6	43.5 ± 21.3	43.2 ± 21.7	19.0–81.0
Height (cm)	178.6 ± 7.2	165.9 ± 7.4	172.0 ± 9.6	155.0–193.0
Weight (kg)	81.5 ± 11.3	66.1 ± 9.6	73.5 ± 12.9	53.0–106.0
BMI (kg/m^2^)	25.6 ± 3.3	24.1 ± 3.7	24.8 ± 3.5	17.7–31.7
Body fat (%)	21.7 ± 7.5	30.0 ± 8.4	26.0 ± 8.4	10.9–41.5

x̅, mean; SD, standard deviation; BMI, body mass index.

Study participants were excluded if they were enrolled in other studies, had any infectious disease, experienced a recent pregnancy or had any metal in their body or any type of cardiac devices.

### Study design

The participants were examined twice within a week. The pre-examination included a medical history, sports activity questionnaire, and weight and height measurements. We then carried out systematic body mapping, and two consecutive caliper and US measurements were taken to evaluate intrarater reliability. Consecutively, a bioelectrical impedance analysis (BIA) was performed. To assess interrater reliability, the second observer repeated the process within a week (median: 2 days). Both measurements were carried out in the morning. During the trial, the participants were instructed not to change their behavior in diet or training and were scheduled independently and randomly by raters. The participants played an exclusively passive role in the measurements, thus there was no learning effect or habituation to the study setting. After the measuring procedure, field 2, 15, 36 and 39 were marked so that the MRI could be done immediately afterwards. MRI measurements were only scheduled for 50 participants.

### Sample size

Sample size was defined by a case number calculation (G-Power Ver. 3.1.9.2). To detect a difference of 10 percent between SFT measurements at 0.8 power, the sample size required is n = 42. We recruited and enrolled 54 participants to ensure sensitivity.

### Mapping

**Figure 1:**
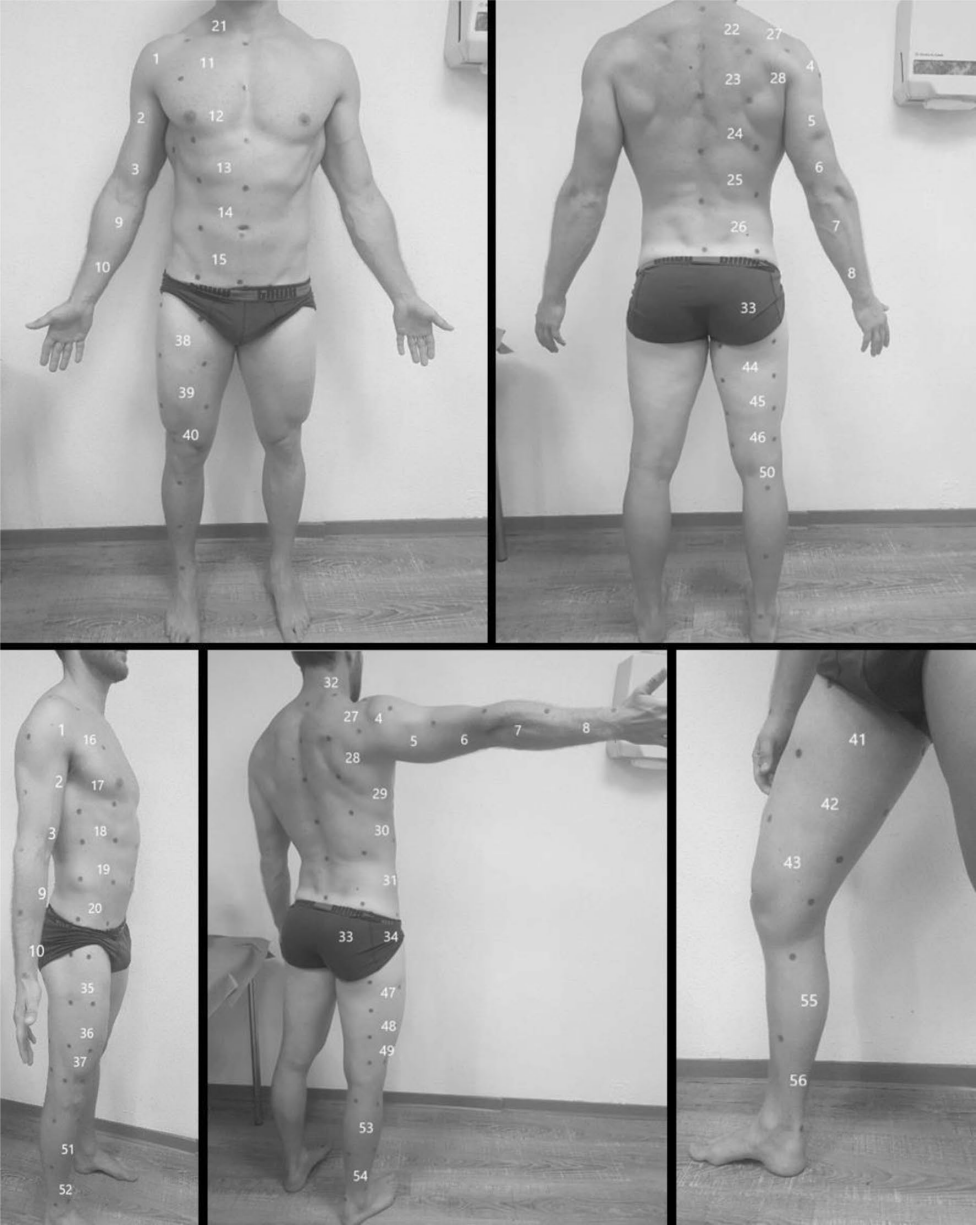
Body Mapping landmarks.

The emphasis of our study is to measure SFT applying a systematic mapping method. On the basis of a pilot trial, we found excellent reliability between left and right side of the body (n = 49, ICC 0.998). Therefore, only the right side of the body was divided into 56 rectangles. The head, hand, foot, and genital areas were omitted. Each rectangle was numbered (1–56, Fig. 1) and marked with adhesive dots on the subject standing. The upper and lower medial length-marking points were first set for each body part. The distance in between was measured, and divided by the number of fields defined and marked along the virtual line. The width of a rectangle was determined by further landmarks or delimited by adjacent fields. For example, fields 47–49 on the posterior thigh have their medial origin at their anatomically defined location and adjoin fields 35–37 on the anterior thigh (Fig. 1, Fields 35–37, 47–49). The center of each field represented the spot of interest. An accurate description of the landmarks is found in Supplementary Table S1. To avoid a labeling error, a preliminary examination was carried out. Only sites 11 and 51 were classified as statistically significant, meaning that the probability of errors occurring is 5% or less.

### Skinfold caliper

The thickness of skinfolds was measured with a Holtain caliper (Holtain, Dyfed, UK, range 0–40 mm) with 0.2 mm accuracy. After mapping, the measurement started with the subject in supine position. Our aim was to measure the skinfold in the center of a field along Langer’s lines. The caliper was applied at right angles to the pinch*.* Within each field, two measurements formed an average value, which was divided by two due to the skinfold double layer. To avoid discomfort and to leave the measurement accuracy unaffected in the presence of glandular tissue, no caliper measurements were taken on fields 12 and 17 in women. Once all the anterior parts had been measured, the subject moved into a prone position.

### US measurements

The US images were generated by a B-Mode device (GE Healthcare GmbH, LOGIQ e, Vivid series) with linear transducers of 12 MHz in longitudinal position to measure SFT depending on the approximate tissue depth. To ensure a primal standardized default setting, the preset ‘musculoskeletal’ (10 MHz) was initially selected. However, an optimum of brightness, gain and dynamic range was individually adjusted by the observers to achieve the best possible tissue delimitability. The measurement area should be aligned to the center of the image.

When ultrasonic gel was applied in the middle of the field, the probe was placed perpendicular in longitudinal position to the inspected tissue, which was ensured by the structure’s optimal brightness and sharpness. When boundaries were clearly distinguishable, the US probe was slowly lifted off until the pressure was low enough due to SFT viscoelasticity. When the area of interest was clearly definable, the image was captured. In abdominal areas, the image was captured when the subject stopped breathing at mid-tidal expiration. As the MRI or the caliper cannot clearly differentiate fibrous structures or the skin, they were not excluded from US either.

The distance between skin and muscle tissue was measured to 0.1 mm accuracy using integrated software tools. Since the caliper involves the skin, it was not excluded from US either.

### MRI

In 50 participants MRI measurements of SFT at 4 sites were performed (fields: 2, 15, 36, 39; Philips Achieva 1.5 T). In 34 of these participants, additional measurements of skinfolds were conducted to visualize the anatomy of field 15. This was done in order to clarify the marked differences between US and Caliper in field 15, which revealed the highest incongruence (51.98%). The gradient strength of the system was 33 mT/m with a maximum slew rate of 122 T/m/s. A whole body coil was selected. The cross-sectional image was displayed after fields were marked with a specific pellet (Fig. 4), which was visible on the skin surface in T1- and T2-weighting due to its water content (MRI protocol Supplementary Table S2). As the Caliper is incompatible with MRI, the test person held the marked skinfold in field 15 with their own hands after receiving detailed instructions. A sagittal slice was obtained while the subject was in supine position. Although we were aware that the skinfold pressure would vary individually, this idea was primarily used to elucidate the difference between US and Caliper.

### Bioelectrical impedance analysis (BIA)

Only for descriptive purposes, TBF was determined via Body Comp Software 8.5 Professional (MEDICAL HealthCare GmbH, www.medi-cal.de) using the segmental BIA 101 Anniversary Sport Edition (Akern srl, Florence, Italy). SFT and TBF measurements were carried out consecutively.

### Statistical analysis

All statistical evaluations are done with the programs SPSS 23 (SPSS Inc., Illinois, USA) and GraphPad Prism 9.1.1 (GraphPad Software Inc., California, USA, www.graphpad.com). Arithmetic mean (x̅), standard deviation (SD), standard deviation of difference (s_D_) and mean difference (d̅) are calculated for descriptive statistics. The Intraclass coefficient (ICC; absolute agreement, single measures) was used as the primary quality and 95% confidence interval (CI), respectively. The 95%CI classifications of the ICC: criterion for reliability ≤ 0.5 poor reliability, 0.5–0.75 moderate reliability, 0.75–0.9 good reliability and values greater than 0.90 indicate excellent reliability^14^. The two-way random effects model was used to calculate the ICC of interrater reliability and two-way mixed effects for intrarater reliability^14^. To visualize test differences, a Bland–Altman plot illustrated the results^15^. The MRI image was analysed via JiveX’s software of Visus Health IT GmbH (2020–2022; www.visus.com).

## Results

We evaluated 53 participants for caliper and 54 for US measurements applying our mapping method. One had to be excluded for caliper as his skinfold measurement was not applicable during the measuring process.

### Intrarater reliability

**Figure 2:**
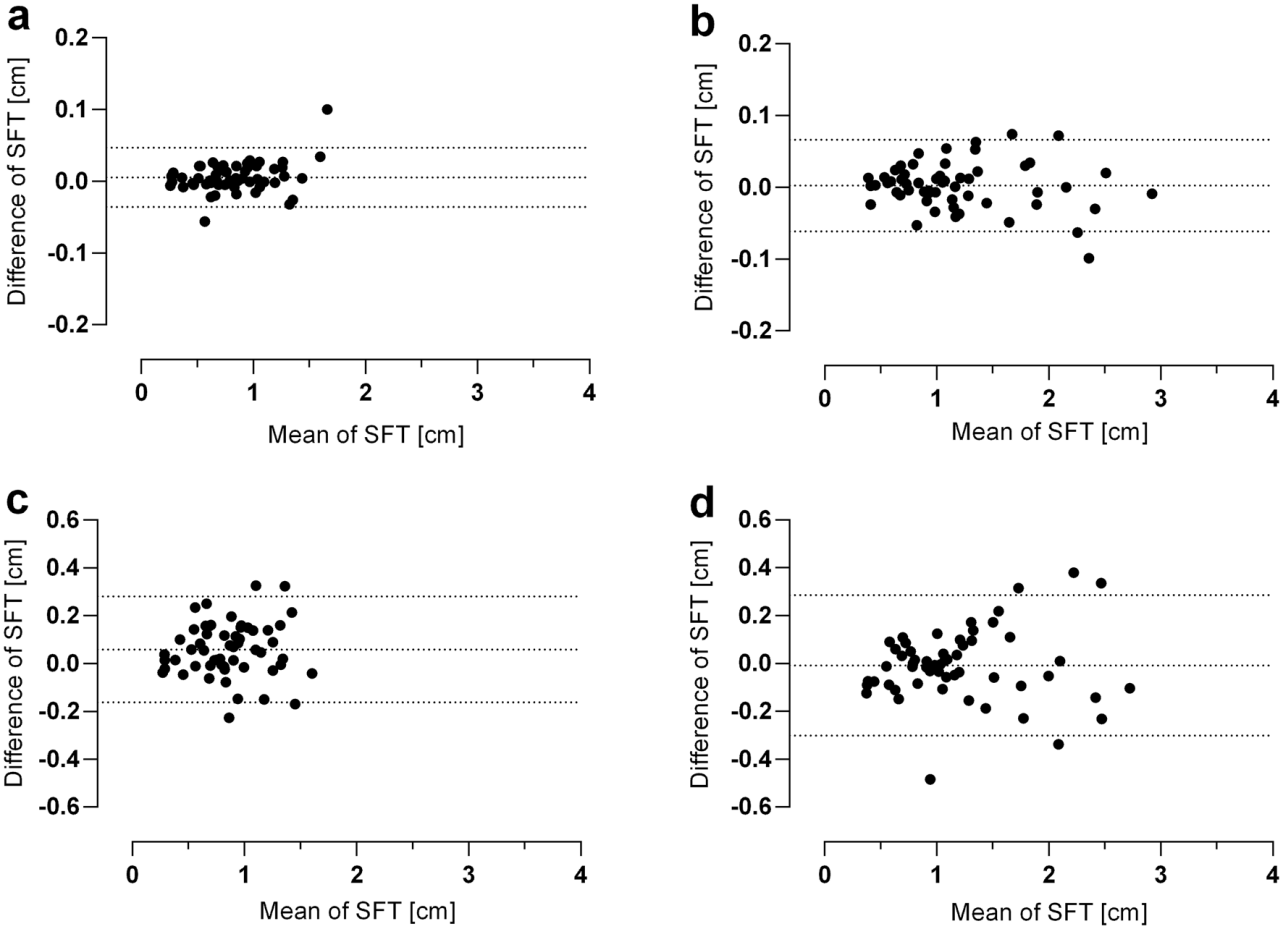
Bland–Altman-Plot (**a**): intrarater caliper reliability (mean:-0.01 ± 0.02 cm; 95% limits of agreement − 0.04 to 0.05 cm); (**b**): intrarater US reliability (mean: 0.00 ± 0.03 cm; 95% limits of agreement of − 0.062 to 0.07 cm); (**c**): interrater caliper reliability (mean: 0.06 ± 0.11 cm; 95% limits of agreement of − 0.16 to 0.28 cm); (**d**): interrater US reliability (− 0.01 ± 0.15 cm; 95% limits of agreement of − 0.30 to 0.29 cm); SFT, subcutaneous fat tissue.

Out of 56, 55 sites measured by caliper (n = 53) and all of US (n = 54) displayed an ICC higher than 0.75 (Supplementary Tables S3 and S4). The caliper’s 95%CI of ICC values ranged from 0.93 to 0.96 and for US from 0.92 to 0.94. The mean difference of each caliper-measured field was − 0.01 ± 0.02 cm (Fig. 2a) and − 0.02 ± 0.03 cm for US (Fig. 2b). There were no systematic errors in these measurements.

### Interrater reliability

The first observer is a highly experienced sonographer (5 years of musculoskeletal US and caliper), whereas the second observer was trained (4 months US and caliper) until his US images met the qualitative standard: (a) optimum of brightness, gain and depth, (b) tissue aligned to the center of the image, (c) distinguishable boundaries, (d) adequate echogenicity of inspected tissue, (e) detecting and minimizing artifacts, (f) reproducible image. Fourtythree participants were engaged for the interrater assessment. Another participant for caliper was excluded because of difficulties during the measuring process.

The caliper’s interrater reliability revealed 14 areas with an ICC beneath 0.75 (Supplementary Table S5). The posterior thigh and calf proved to be the poor-to-moderately-reliable areas most often, whereas the chest/abdomen, anterior thigh and back were very reliable. The 95%CI of ICC values ranged from 0.754 to 0.835. Figure 2c represents the Bland–Altman analysis showing SAT differences between the two caliper measurements plotted against the mean. It illustrates a mean of difference of 0.05 ± 0.11 cm. In comparison, US had 10 of 56 sites showing an ICC lower than 0.75. Especially the chest/abdomen, anterior and posterior thigh and calf were very reliable. The lower arm on the other hand was not reliable. The 95%CI of ICC ranged from 0.794 to 0.857 (Supplementary Table S6). Figure 2d represents US’s Bland–Altman plot with a mean difference of − 0.01 ± 0.15 cm.

### Comparison of methods

The comparison of MRI and US revealed an overall average difference of − 0.13 ± 0.34 cm and an excellent reliability (0.948). The values for the different measuring sites are given in Table 2.

**Table 2:** SFT depth comparison of MRI, US and Caliper at 4 sites.

MRI vs. US (n = 50)	MRI	US	d	ICC
Region (field)	Mean ± SD (cm)			
Bizeps (2)	0.62 ± 0.35	0.82 ± 0.52	− 0.20 ± 0.38	0.593
Abdomen (15)	2.52 ± 1.26	2.71 ± 1.34	− 0.19 ± 0.41	0.942
Mid front thigh (36)	1.15 ± 0.62	1.20 ± 0.62	− 0.06 ± 0.21	0.938
Mid lateral thigh (39)	1.03 ± 0.75	1.09 ± 0.78	− 0.06 ± 0.31	0.891

MRI vs. Caliper (n = 50)	MRI	Caliper	d	ICC
bizeps (2)	0.62 ± 0.35	0.54 ± 0.35	0.08 ± 0.27	0.688
abdomen (15)	2.52 ± 1.26	1.26 ± 0.61	1.26 ± 0.80	0.374
mid front thigh (36)	1.15 ± 0.62	1.07 ± 0.54	0.07 ± 0.32	0.849
mid lateral thigh (39)	1.03 ± 0.75	1.01 ± 0.66	0.02 ± 0.41	0.835

US vs. C (n = 50)	US	Caliper	d	ICC
bizeps (2)	0.82 ± 0.52	0.54 ± 0.35	0.28 ± 0.31	0.633
abdomen (15)	2.71 ± 1.34	1.26 ± 0.61	1.45 ± 0.90	0.318
mid front thigh (36)	1.20 ± 0.62	1.07 ± 0.54	0.13 ± 0.28	0.862
mid lateral thigh (39)	1.09 ± 0.78	1.01 ± 0.66	0.08 ± 0.35	0.879

Comparison of subcutaenous fat depth (cm) between devices. MRI, magnetic resonance imaging; US, ultrasound; SD, standard deviation; ICC, intraclass coefficient; d, mean difference; n, number of participants.

**Figure 3:**
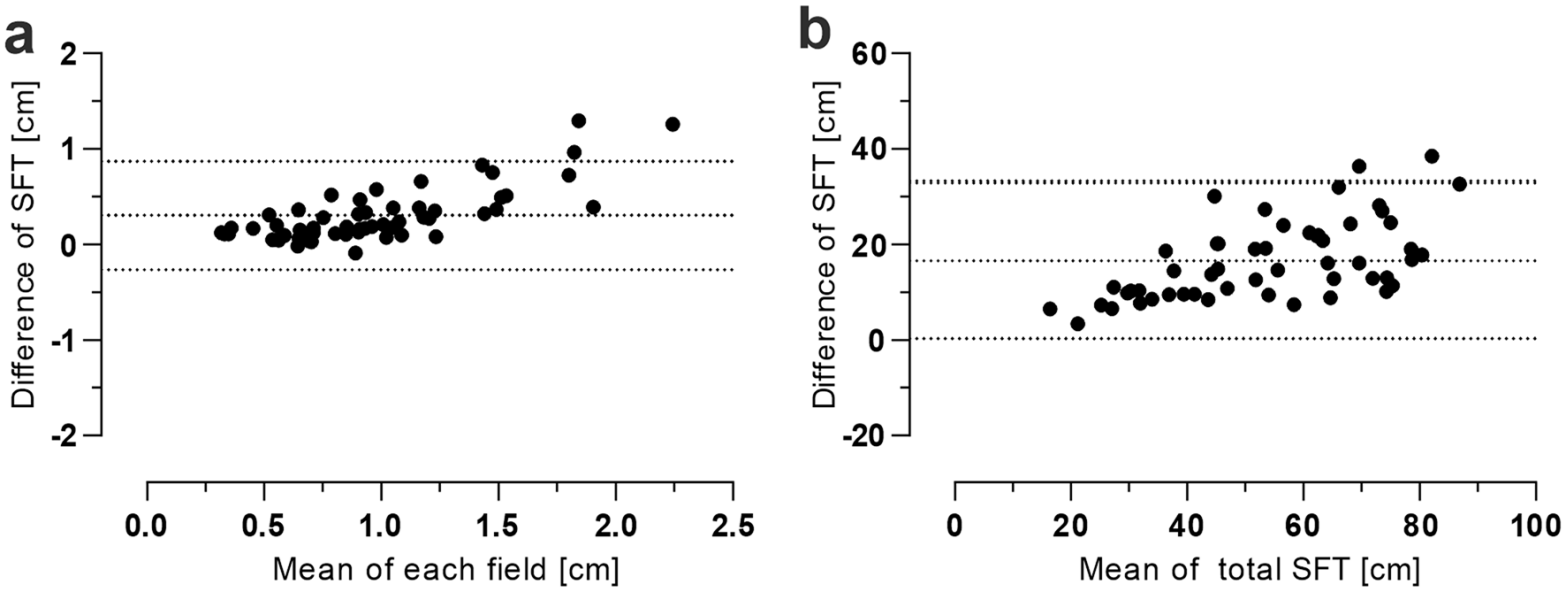
Caliper vs. US Bland–Altman-Plot: (**a**): mean of SFT depth of each field (1–56); mean: 0.3 ± 0.29 cm with 95% limits of agreement of − 0.26 to 0.87 cm. (**b**): Cumulated SFT of all fields (total SFT) of each subject (n = 53, field 12 and 15: only men); mean: 16.61 ± 8.31 cm with 95% limits of agreement 0.32–32.90 cm.

Our comparison of caliper to US at 56 sites demonstrated clear differences in mean SAT depth of all areas (0.83 ± 0.33 cm vs. 1.14 ± 0.54 cm; p < 0.001, n = 53) and moderate reliability (ICC 0.669, 95%CI: 0.62–0.71). Figure 3a represents the Bland–Altman plot of every subject’s field with a mean difference of 0.3 ± 0.29 cm. Field 15 revealed the greatest mean deviance (− 1.29 ± 0.76 cm (51.98%; ICC: 0.416)), whereas the medial back (field 23–26) and anterior lower leg (field 51–52) were very reliable (Supplementary Table S7). To enable an overview of the total subcutaneous fat depth, all fields in each subject were cumulated (total SFT), as shown in Fig. 3b. The mean value of the total SFT difference between caliper and US was 16.61 ± 8.31 cm (26.86%; ICC: 0.581), indicating that caliper measures clearly less tissue than US*.* The two graphs illustrate remarkably the difference between these methods, particularly when there is more subcutaneous body fat.

**Figure 4:**
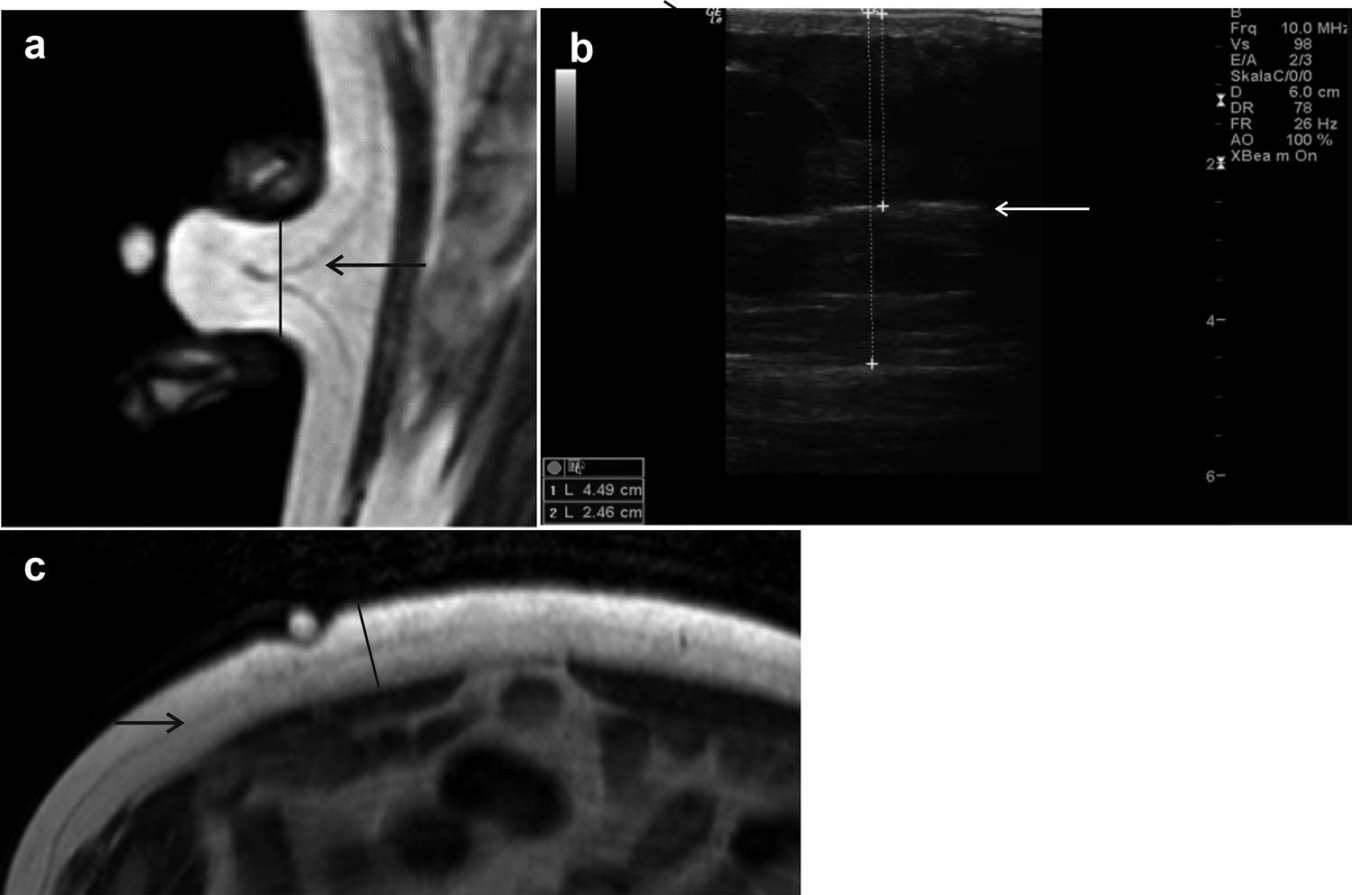
Field 15 (**a**): MRI sagittal slice (SI) of lower abdomen when subject held skinfold to explain the difference between US and caliper SFT values. The line represents the caliper’s measuring point. (**b**): US image measured with a 12 Hz probe showing a 4.49 cm SFT depth. (**c**): MRI transversal image shows the pellet marked at field 15. The line illustrates SFT depth. The arrow indicates Scarpa’s fascia in all images.

Considering the difference in SFT between caliper and US, especially at abdominal field (#15), a sagittal MRI (SI) was done to detect discrepancies. Thirty four participants from the total MRI sample were evaluated. Table 3 shows field 15’s subcutaneous fat depth values measured by MRI, US (Fig. 4b) and caliper. The MRI images contain two measurements: (1) native, transversal image (MRI (TI)) to show the mean value of the subcutaneous fat depth (Fig. 4c); (2) the sagittal image (MRI Skinfold (SI), Fig. 4a) displaying the subcutaneous fat layer held with hands in measuring position for caliper.

**Table 3:** Depth of field #15 SFT as measured by MRI (SI), MRI (TI), US and caliper (abdominal area).

n = 34	MRI (TI) (cm)	US (cm)	Caliper (cm)	MRI Skinfold (SI) (cm) analogous to caliper measurement (Fig. 4a)
Mean ± SD	2.26 ± 1.32	2.43 ± 1.36	1.15 ± 0.66	0.96 ± 0.61
ICC	0.966		0.880	
		0.429		
	0.469			

MRI, magnetic resonance imaging; TI, transversal image; SI, sagittal image (fat layer held with hands in measuring caliper position); US, ultrasound; SD, standard deviation; ICC, intraclass coefficient; SFT, subcutaneous fat tissue. The measured caliper values were divided by two because of the double fat layer. SI, sagittal image; TI, transversal image; n, number of participants (skinfold measurements were performed in 34 of 50 participants).

The interrater reliability at 8 standardized sites of ISAK is shown in Table 4.

**Table 4:** Interrater reliability compared to recommended ISAK sites.

ISAK skinfolds	#Field	Caliper	US	Caliper vs. US
Biceps	2	+	+	−
Triceps	5	+	+	=
Iliac crest	31	+	+	+
Subscapular	24	+	+	+
Calf	55	=	+	=
Thigh	39	+	+	+
Abdominal	14	−	+	=
Supraspinal	20	+	+	−

+, good to excellent interrater reliability (ICC 0.75–0.9); =, moderate reliability (ICC 0.5–0.75); −, poor interrater reliability (ICC ≤ 0.5).

ISAK, International Society for the Advancement of Kinanthropmetry; US, ultrasound.

## Discussion

Our study’s main finding is the significant difference in fat layer thickness as measured by caliper versus US at most sites despite both methods’ high intra- and interrater reliability. In the abdominal region, the sagittal MRI slice shows that the deep subcutaneous fat layer, below Scarpa’s fascia, is not completely incorporated within the skinfold. The caliper’s accuracy is therefore limited in this area. For two dimensional SFT thickness measurements US is comparable to MRI measurement.

### Caliper’s Intrarater reliability

All 56 caliper sites displayed good to excellent reliability (ICC 95% CI: 0.926–0.963). Wagner et al.^16^ obtained similar results investigating the caliper method’s intrarater reliability and interrater reliability in 45 college athletes. Measurements were taken on the chest, abdomen, and thigh in the men, and on the triceps, upper thigh, and suprailiac in the women. ICC values of 0.996 (95% CI: 0.993–0.998) revealed excellent intrarater reliability. Pérez-Chirinos Buxade et al.^17^ investigated intrarater reliability in 48 participants of different fitness levels. Two raters took measurements at the triceps, subscapular, biceps, iliac crest, supraspinal, abdominal, front thigh and medial calf^17^. The skinfolds’ ICC exceeded 0.989, which is nearly identical with this study’s.

### Intrarater reliability B-mode US

US showed good to excellent intrarater reliability at all areas (ICC 95% CI: 0.924–0.944). Another study achieved similar results at eight sites, namely the upper abdomen, lower abdomen, erector spinae, distal triceps, brachioradialis, lateral thigh, front thigh and medial calf^18^. However, their Spearman correlation coefficient (ρ) amounted to 0.978–0.990. Weiss^19^ investigated the intrarater reliability of B-mode ultrasound in 30 college students. Images were taken at clearly defined locations on the front and back of the right thigh. Their result showed a correlations coefficient of r = 1.00 for men and r = 0.99 for women^19^ using the Spearman correlation. Chandler et al.^20^ assessed US intra- and interrater reliability at seven anatomical locations (9 male, 8 female) and showed strong intrarater reliability (rater 1: ICC: 0.998; 95% CI 0.996–0.999; rater 2: ICC: 0.997; 95% CI 0.992–0.999). Störchle et al.^18^ investigated eight ISAK sites (International Society for the Advancement of Kinanthropmetry) and confirmed a correlation coefficient of p = 0.999 (p < 0.01).

When measuring total SFT, US exhibited smaller overall intrarater differences although it is particularly sensitive since it measures quite punctiformly, while caliper captures a larger area. Disparities may arise by more or less compression or greater fat fluctuations within a field. As a result, both methods identify changes in fat distribution in sites when determined by the same observer.

### Caliper’s interrater reliability

Regarding the caliper, 42 sites showed a good ICC above 0.75 (95%CI: 0.754–0.835). The posterior thigh and calf tended especially toward poor to moderate reliability. Pérez-Chirinos Buxade et al.^17^ investigated interrrater reliability also, detecting a 0.999 ICC with a 95% CI of 0.995–0.999, however, they only measured 8 sites.

Kispert and Merrifield^21^ measured sites in male (triceps, chest, and subscapular) and female participants (triceps, abdomen, and iliac crest). They mentioned that their male participants’ ICCs were higher (ICC: 0.80–0.85) than their female participants (0.62–0.75) due to women’s greater amount of body fat^21^. However, we only analyzed statistics of the entire cohort.

The ISAK considers 8 sites as standard: biceps, triceps, iliac crest, subscapular, supraspinale, calf, front thigh and abdominal^10^. Table 4 shows those fields containing ISAK’s skinfolds although they were not specifically pinpointed. According to our results, six ISAK sites were very reliable; only the abdominal and medial calf revealed moderate to poor caliper reliability. Hume and Marfell-Jones^22^ obtained similar findings, but considered the biceps and triceps also as critical, which we could not confirm in this study. Gonzáles-Ruíz et al.^23^ also confirmed different caliper values at the triceps even with minor changes in location. When the caliper is aligned with Langer’s lines, the point of application may shift between two examiners, also through the labeling procedure. There is evidence that even the smallest shift (one centimeter) can influence the depth measurement value in 70% of sites^22^. Durnin and Womersley^7^ reported more variability in measured values in persons with a higher percentage of body fat. Their finding is evidence of many skinfold formulas, especially considering the relationship between subcutaneous fat and body fat mass.

### Interrater reliability B-mode US

Our US results showed a 95%CI of the ICC of 0.793–0.857, although 11 sites were poor to moderately reliable (0.378–0.744)_,_ but not restricted to a certain area, except for the lower arm. Störchle et al.^18^ discussed the highest absolute deviations at the lower abdomen and lateral thigh, which this study did not confirm. All ISAK sites revealed good to excellent interrater reliability. Müller et al.^24^ investigated 19 female athletes (BMI: 21.3 ± 2.3 kg/m^2^) via US at eight standardized sites and obtained an ICC above 0.9 (exception biceps: ICC 0.87). Chandler et al.^20^ observed strong interrater reliability (ICC: 0.983; 95% CI 0.946–0.994) at seven locations, which we verified in this study as well.

The greatest difficulties with ultrasound measurements occur when determining the fat-muscle transition, especially when a deep fat layer is present^24^. Hoyos et al.^25^ showed that the fat tissue in the posterior upper arm consists of two layers, meaning greater potential for fluctuations during measurements. This can be particularly important in areas with unevenly distributed fat layers such as the gluteal femoral, abdominal, and paralumbar areas^26^. Breathing also affects abdominal SFT measurements, so special caution is required there also^18^.

In conclusion, with increasing SFT, the absolute scatter for US increases, but the relative deviation remains constant. In comparison, the caliper’s absolute deviation is smaller, but it also fails to reach same SFT depth as US.

### Comparison of methods

#### MRI vs US (4 fields)

MRI and US showed very good to excellent reliability except for field 2 (0.593, biceps). The overall difference of mean between both devices is − 0.13 ± 0.34 cm. US measures were systematically higher than MRI. A moderate ICC for biceps may be explained by the US’s higher resolution compared to MRI. Additionally, the narrow space in the MRI device can also affect the results, particularly when the arms must be hold very close to the body and the fat layer is moved. As Störchel et al.^10^ stated, US is the most accurate method for subcutaneous fat depth measurement. MRI (TI) vs. US showed an ICC of 0.966 (2.26 cm vs. 2.43 cm) at the abdominal area. Mechelli et al.^27^ obtained the same results. They confirmed US imaging and MRI measurements of SFT, showing excellent agreement regarding the SFT in the anterior thigh (r = 0.99, p < 0.01, MRI 0.99 ± 0.47 cm; US 1.05 ± 0.47 cm).

#### MRI vs Caliper (4 fields)

Except for field 2 and 15, both methods show a good reliability at mid front thigh and mid lateral thigh (see Table 2). The apparent difference in field 15 (2.51 vs 1.26 cm, ICC 0.374) is potentially caused by mechanical or even histological factors. To investigate the theory, the participant produced a skinfold in field 15 in supine position, because the caliper itself is incompatible with the magnetic field. Although individual hand pressures of skinfolds vary, MRI (SI) data resembled the Caliper values (1.15 cm vs. 0.96 cm, ICC: 0.880). The human trunk’s subcutaneous fat tissue consists of two layers separated by Scarpa’s fascia: the superficial fat layer (SFL) and deep fat layer (DFL)^28^. Considering the US and transversal MRI (TI) image (Fig. 4), note that Scarpa’s fascia divides the SFT at this area into nearly half for this study population, forming a so-called “lambda-sign” indicating non-inclusion of the DFL within the caliper. Hence, only the skinfold’s SFL will be integrated twice. If the caliper value is then divided by two, it will only display the SFL in the abdominal area, thus explaining the approximate 50% difference between caliper and US. There are of course differences, depending on gender, amount of body fat, its distribution and location^29^. Harley and Pickford^29^, on the other hand, showed that the mid-abdomen’s DFL is thicker than the lower abdomen’s. Female participants are much more likely to present a thicker fat layer in the abdomen up to the umbilicus than is detected in most of the inferior area^29^. If the DFL is not incorporated within the caliper at that area, both layers must be relocatable. Lancerotto et al.^30^ investigated the structure of the abdominal SAT microscopically. They found that the SFL consists of large fat lobes organized in single or multiple layers. Fibrous septa encased the fat lobes like in a honeycomb, and were positioned consistently and perpendicularly to the dermis. Furthermore, these septa connected the deep dermis to Scarpa’s fascia, and consisted of collagen and elastic fibers^30^. Their study confirmed that the SFL possesses highly stable and elastic components, while the honeycomb fat lobes return to their initial position after relocation, thus contributing to the SAT’s mechanical equilibrium. On the contrary, they found that the DFL consists of fat lobes being smaller and arranged in a less well-structured pattern^30^. The fibrous septa, however, are more obliquely-horizontally aligned, and there are few elastic components. Lateral displacement was easily realized, but the original position was inconsistently regained. Lancerotto et al.^30^ stated that, therefore, the two fat layers have a “sliding system”, meaning that when SFL is pulled up by hands, the DFL clings primarily to the deep fascia. This appears to clarify the displacement of SFL and DFLs when taking caliper measurements. Similarly, thigh and gluteal areas also possess these two layers, a factor that needs to be considered when using a caliper^30^.

#### US vs Caliper (56 fields)

Our results for all 56 fields of caliper and ultrasound measurements revealed that 38 sites (67%) were below an ICC of 0.75 (ICC 0.699, 95% CI: 0.625–0.712). The two devices were equivalent only in the lower medial and lateral back. Caliper measures 24.31% less SFT on average over all fields than US (mean of field differences, see Supplementary Table S7). Higher SFT values for US were already measured by Kuczmarski et al.^31^. Akyer et al.^32^ and Selkow et al.^33^, however, they detected lower values with US conducted at ISAK sites. The compression of skin and fat tissue can result in lower measured values^24^. Determining the fat-muscle transition becomes difficult also when fibrous structures are embedded in the muscle. Concerning the ISAK, in this study we found that only the subscapular area and anterior thigh proved to show good to excellent reliability in caliper-to-US comparisons. While taking these measurements, we noticed that the caliper and US skinfold values differed tremendously at certain parts of the body. Therefore, which sites function equivalently must be clearly defined, especially when body-fat analyses are relying on caliper measurements. Compression seems to play some kind of a role in such differences between these methods as the caliper compresses fat tissue more than US does. The abdomen area especially (field 15) showed a 51.98% (Supplementary Table S7) SFT depth difference between the two methods. Measurement-value deviations differ in only one direction despite including a wide range of body types. In particular, the abdominal area, front and upper lateral thigh, and lower arm displayed differences of 25–50%. Due to the non-inclusion of the DFL into the caliper at the abdominal area, sites consisting of a double layer should be interpreted with caution.

To ensure comparability among studies, a standardized protocol should be adopted that relies on both reliability and validity data for statistical analyses (i.e. ICC, coefficient of variance).

### Limitations

This mapping method of ours includes 56 measuring points conceived as specific landmarks. Note that the measurements were taken in one session relying on previous markings. During pre-examinations, the repeated marking process showed only a 5% maximum difference. Even if the labeling process revealed no mean differences between observers, it can still affect the variance. Since the body is rather cone-shaped, rectangles are arranged somewhat inhomogeneously. Furthermore, a standard routine for taking ultrasound and caliper measurements is imperative to ensure reliability. Such examinations should only be conducted by experienced sonographers. The exact balance between the ultrasound probe’s lift-off and freezing the image varies individually. Handling these instruments requires adequate qualification. Furthermore, the ISAK sites we relied on were not pinpointed, but were nevertheless incorporated within the measurement area. These results allow only a statement for the included study population. For validity conclusions, a higher sample size is required.

## Conclusion

As measuring subcutaneous fat via US or caliper yields significant differences at most areas, the two methods are not interchangeable. Caliper drastically underestimates the depth of subcutaneous fat tissue depending on its location. Both measurement methods yield very good intrarater data and relative changes can be identified. Only the chest/abdomen and anterior thigh are interrater-reliable with both devices. In direct comparisons, the lower medial and lateral back deliver equivalent results. Regarding ISAK skinfolds, the Iliac crest, subscapular and thigh can be measured interchangeably and equally reliably via caliper or US. The calf, on the contrary, is only measured reliably via US. However, to obtain accurate SFT depth measurements, ultrasound is the method of preference as it captures all fat layers most precisely. When compared to MRI, US is more readily available in the daily practice, although both methods yield similar results. Subcutaneous fat tissues consisting of a double fat layer (like the abdomen) must be examined with particular caution, when measuring with a caliper, as it does not measure both fat layers.

## Supplementary Information

Supplementary Tables.

## Data Availability

The original contributions presented in the study are included in the article supplementary material; further inquiries can be directed to the corresponding authors.
