# Supplementary material for: Measurement of subcutaneous fat tissue: reliability and comparison of caliper and ultrasound via systematic body mapping
Source: Sci Rep. 2022 Sep 22;12:15798. doi: 10.1038/s41598-022-19937-4 (PMC9500055; doi:10.1038/s41598-022-19937-4)
Supplement: Supplementary file 1 — Supplementary Tables. [file 41598_2022_19937_MOESM1_ESM.docx]

Supplementary Table S1 Landmark positions

| **Body part** | **Section** | **Field #** | **Landmarks** |
| --- | --- | --- | --- |
| **Upper Arm** | Biceps | 1-3 | Length: Acromion up to epicondylus radialis  Width 1: pars clavicularis (M. deltoideus)  Widths 2+3: Sulcus bicipitalis medialis (ventral) |
|  | Triceps | 4-6 | Length: Acromion up to epicondylus radialis  Width 4: pars sternalis (M. deltoideus)  Width: 5+6: Sulcus bicipitalis medialis (dorsal) |
| **Lower Arm** | lateral | 7-8 | Length: Epicondylus radialisup to distal head of radius  Width: half circumference lower arm (dorsal) |
|  | medial | 9-10 | Lengh: Epicondylus radialis to distal head of radius  Width: half circumference lower arm (palmar) |
| **Chest/Abdomen** | medial | 11-15 | Length: vertical line through Incisura jugularis to level of SIAS  Width: up to vertical line through SIAS |
|  | lateral | 16-20 | Length: Clavicula to SIAS (parallel to median line)  Width: up to mid-axillary line (# 16: If necessary, only up to the anterior axillary line) |
|  | anterior neck | 21 | Length: Chin to incisura jugularis  Width: Incisura jugularis to perpendicular line through base of ear |
| **Back** | medial | 22-26 | Length: C7 to level of SIPS  Width: Midpoint between SIPS and mid-axillary line |
|  | lateral | 27-31 | Length: above the midpoint between SIPS and mid-axillary line at the level of medial line  Width: up to mid-axillary line, #27 to posterior axillary line |
|  | posterior neck | 32 | Length: Os occipitale to C7  Width: horizontal line through C7 up to vertical line through ear base |
| **Gluteal Region** | medial | 33 | Length: lowest point of posterior median line to medial gluteal fold  Width: medial to lateral end of the gluteal fold |
|  | lateral | 34 | Length: Level of SIPS to the gluteal fold  Width: end of gluteal fold up to trochanter major |
| **Anterior Thigh** | lateral | 35-37 | Length: vertical line: the greater trochanter to the level of the upper edge of the patella  Width: up to lateral patella pole |
|  | medial | 38-40 | Length: along M. vastus lateralis at level of lateral patella pole  Width: to M. vastus medialis muscle at level of medial patella pole |
|  | adductors | 41-43 | Length: along M. vastus medialis muscle at level of medial patella pole  Width: to medial posterior thigh |
| **Posterior Thigh** | medial | 44-46 | Length: medial buttock contour to distal end of semimembranosus/tendinosus muscles (at level of patella’s upper edge)  Width: to the vertical line: end of the gluteal fold to the level of the patella pole |
|  | lateral | 47-49 | Length: lateral buttock contour to distal end of biceps femoris muscle (at level of patella’s upper edge)  Width: up to the vertical line of the greater trochanter |
| **Knee** | popliteal fossa | 50 | Length: level of patella’s upper edge to the tibial tuberosity  Width: femurepicondyles from medial to lateral |
| **Anterior Lower Leg** | M. tibialis  anterior | 51-52 | Length: tuberositas tibiae to ankle joint crease  Width: up to malleolus lateralis line |
| **Calf** | M. gastrocnemius  lateralis | 53-54 | Length: Level of tibial tuberosity to Achilles tendon  Width: to malleolus lateralis line |
|  | M. gastrocnemius  medialis | 55-56 | Length: Level of tibial tuberosity to Achilles tendon Width: to malleolus medialis line |

SIAS: spina iliaca anterior superior, SIPS: spina iliaca posterior superior

Supplementary Table S2 MRI protocol for skinfold measurement (field 15)

| Field 15 | Sequenztyp | Slice thickness (mm) | | TR  (ms) | TE  (ms) | flip | time  (m:s) |
| --- | --- | --- | --- | --- | --- | --- | --- |
| transversal | TSE T1w | | 8 | 350 | 10 | 90 | 00:15 |
| transversal | SSh-TSE T2w | | 10 | 818 | 80 | 90 | 00:12 |
| sagittal | SSh-TSE T2w | | 10 | 818 | 80 | 90 | 00:12 |
| sagittal | TSE T1w | | 8 | 350 | 10 | 90 | 00:57 |

T1w: T1-weighted, T2w: T2-weighted, Flip: Flipangle, SSh-TSE: single shot-turbo spin echo sequence, m: minutes, s: seconds

Supplementary Table S3 Caliper’s Intrarater Reliability (n=53)

| **Body part** | **Field**  **#** | **C1**  **(cm)** | **C2**  **(cm)** | **d̅**  **(cm)** | **S_D_**  **(cm)** | **ICC** |
| --- | --- | --- | --- | --- | --- | --- |
| Upper Arm | 1 | 0.54 | 0.52 | 0.02 | 0.07 | 0.959 |
|  | 2 | 0.47 | 0.47 | 0.00 | 0.09 | 0.962 |
|  | 3 | 0.37 | 0.38 | -0.01 | 0.11 | 0.866 |
|  | 4 | 0.96 | 0.94 | 0.02 | 0.18 | 0.917 |
|  | 5 | 0.91 | 0.90 | 0.00 | 0.12 | 0.955 |
|  | 6 | 0.61 | 0.63 | -0.02 | 0.11 | 0.956 |
| Lower Arm | 7 | 0.29 | 0.28 | 0.01 | 0.07 | 0.935 |
|  | 8 | 0.27 | 0.28 | 0.00 | 0.04 | 0.962 |
|  | 9 | 0.37 | 0.36 | 0.00 | 0.06 | 0.962 |
|  | 10 | 0.26 | 0.26 | -0.01 | 0.05 | 0.956 |
| Chest/Abdomen | 11 | 0.69 | 0.67 | 0.02 | 0.15 | 0.928 |
|  | 12* | 1.14 | 1.19 | 0.00 | 0.14 | 0.985 |
|  | 13 | 0.97 | 0.97 | 0.00 | 0.10 | 0.988 |
|  | 14 | 1.10 | 1.10 | 0.00 | 0.09 | 0.991 |
|  | 15 | 1.20 | 1.18 | 0.02 | 0.08 | 0.992 |
|  | 16 | 0.53 | 0.51 | 0.02 | 0.09 | 0.969 |
|  | 17* | 0.82 | 0.86 | -0.02 | 0.11 | 0.986 |
|  | 18 | 0.74 | 0.72 | 0.02 | 0.08 | 0.989 |
|  | 19 | 0.77 | 0.75 | 0.01 | 0.11 | 0.974 |
|  | 20 | 0.67 | 0.67 | 0.00 | 0.08 | 0.977 |
| Anterior Neck | 21 | 0.65 | 0.63 | 0.03 | 0.15 | 0.886 |
| Back | 22 | 0.27 | 0.27 | 0.01 | 0.06 | 0.887 |
|  | 23 | 0.93 | 0.92 | 0.01 | 0.10 | 0.983 |
|  | 24 | 0.69 | 0.69 | -0.01 | 0.07 | 0.984 |
|  | 25 | 0.80 | 0.80 | 0.00 | 0.06 | 0.992 |
|  | 26 | 0.85 | 0.84 | 0.00 | 0.11 | 0.964 |
|  | 27 | 1.07 | 1.04 | 0.03 | 0.14 | 0.969 |
|  | 28 | 0.83 | 0.83 | -0.01 | 0.13 | 0.940 |
|  | 29 | 0.84 | 0.84 | 0.00 | 0.12 | 0.974 |
|  | 30 | 0.76 | 0.75 | 0.01 | 0.08 | 0.979 |
|  | 31 | 0.75 | 0.75 | 0.00 | 0.11 | 0.983 |
| Posterior Neck | 32 | 1.34 | 1.37 | -0.03 | 0.16 | 0.949 |
| Gluteal | 33 | 1.71 | 1.61 | 0.10 | 0.34 | 0.811 |
|  | 34 | 1.62 | 1.58 | 0.03 | 0.32 | 0.895 |
| Anterior Thigh | 35 | 1.27 | 1.25 | 0.02 | 0.09 | 0.990 |
|  | 36 | 0.98 | 0.95 | 0.03 | 0.08 | 0.990 |
|  | 37 | 0.67 | 0.66 | 0.01 | 0.09 | 0.973 |
|  | 38 | 1.05 | 1.06 | -0.01 | 0.13 | 0.962 |
|  | 39 | 1.04 | 1.03 | 0.00 | 0.13 | 0.971 |
|  | 40 | 0.96 | 0.93 | 0.02 | 0.12 | 0.926 |
|  | 41 | 1.01 | 1.03 | -0.02 | 0.15 | 0.965 |
|  | 42 | 1.28 | 1.25 | 0.03 | 0.12 | 0.962 |
|  | 43 | 1.28 | 1.27 | 0.01 | 0.14 | 0.969 |
| Posterior Thigh | 44 | 1.31 | 1.34 | -0.03 | 0.14 | 0.967 |
|  | 45 | 0.87 | 0.87 | 0.00 | 0.13 | 0.957 |
|  | 46 | 0.65 | 0.67 | -0.02 | 0.14 | 0.882 |
|  | 47 | 1.44 | 1.43 | 0.00 | 0.14 | 0.982 |
|  | 48 | 1.04 | 1.02 | 0.02 | 0.19 | 0.952 |
|  | 49 | 0.86 | 0.84 | 0.02 | 0.21 | 0.899 |
| Fossa poplitea | 50 | 0.45 | 0.46 | 0.00 | 0.09 | 0.932 |
| Anterior Lower Leg | 51 | 0.51 | 0.51 | 0.00 | 0.07 | 0.958 |
|  | 52 | 0.54 | 0.60 | -0.06 | 0.31 | 0.536 |
| Calf | 53 | 0.62 | 0.61 | 0.00 | 0.07 | 0.971 |
|  | 54 | 0.58 | 0.58 | 0.00 | 0.08 | 0.959 |
|  | 55 | 0.62 | 0.63 | 0.00 | 0.08 | 0.954 |
|  | 56 | 0.79 | 0.79 | 0.00 | 0.20 | 0.829 |
|  | **SF_Total_ Depth** | 45.63 | 45.28 | 0.31 | 1.38 | 0.996 |

SF_Total_ Depth: total depth of subcutaneous fat, S_D_: standard deviation of difference, d̅: mean of difference, ICC: Intraclass coefficient; C: caliper, *only male subjects n=28

Supplementary Table S4 US Intrarater Reliability (n=54)

| **Body part** | **Field**  **#** | **US 1 (cm)** | **US 2 (cm)** | **d̅**  **(cm)** | **S_D_**  **(cm)** | **ICC** |
| --- | --- | --- | --- | --- | --- | --- |
| Upper Arm | 1 | 0.60 | 0.59 | 0.01 | 0.08 | 0.954 |
|  | 2 | 0.84 | 0.84 | 0.01 | 0.19 | 0.928 |
|  | 3 | 0.54 | 0.53 | 0.01 | 0.12 | 0.890 |
|  | 4 | 1.15 | 1.19 | -0.04 | 0.23 | 0.868 |
|  | 5 | 1.14 | 1.17 | -0.03 | 0.15 | 0.961 |
|  | 6 | 0.92 | 0.93 | -0.01 | 0.17 | 0.940 |
| Lower Arm | 7 | 0.41 | 0.41 | 0.00 | 0.09 | 0.906 |
|  | 8 | 0.40 | 0.42 | -0.02 | 0.11 | 0.899 |
|  | 9 | 0.70 | 0.67 | 0.03 | 0.12 | 0.936 |
|  | 10 | 0.40 | 0.38 | 0.01 | 0.11 | 0.916 |
| Chest/Abdomen | 11 | 1.29 | 1.28 | 0.01 | 0.25 | 0.941 |
|  | 12 | 2.16 | 2.16 | 0.00 | 0.35 | 0.968 |
|  | 13 | 1.38 | 1.32 | 0.06 | 0.21 | 0.967 |
|  | 14 | 1.90 | 1.90 | -0.01 | 0.21 | 0.984 |
|  | 15 | 2.52 | 2.50 | 0.02 | 0.20 | 0.988 |
|  | 16 | 1.06 | 1.05 | 0.01 | 0.25 | 0.917 |
|  | 17 | 2.41 | 2.44 | -0.03 | 0.68 | 0.925 |
|  | 18 | 1.09 | 1.06 | 0.03 | 0.19 | 0.969 |
|  | 19 | 1.13 | 1.15 | -0.02 | 0.17 | 0.967 |
|  | 20 | 1.18 | 1.22 | -0.04 | 0.22 | 0.955 |
| Anterior Neck | 21 | 0.64 | 0.62 | 0.02 | 0.11 | 0.930 |
| Back | 22 | 0.45 | 0.45 | 0.00 | 0.14 | 0.812 |
|  | 23 | 0.86 | 0.81 | 0.05 | 0.18 | 0.917 |
|  | 24 | 0.72 | 0.70 | 0.02 | 0.11 | 0.955 |
|  | 25 | 0.90 | 0.92 | -0.02 | 0.19 | 0.934 |
|  | 26 | 1.04 | 1.02 | 0.02 | 0.18 | 0.941 |
|  | 27 | 1.38 | 1.36 | 0.02 | 0.32 | 0.867 |
|  | 28 | 1.00 | 0.99 | 0.01 | 0.20 | 0.888 |
|  | 29 | 0.99 | 0.99 | -0.01 | 0.29 | 0.888 |
|  | 30 | 0.97 | 1.00 | -0.03 | 0.17 | 0.958 |
|  | 31 | 0.89 | 0.89 | -0.01 | 0.15 | 0.965 |
| Posterior Neck | 32 | 2.31 | 2.41 | -0.10 | 0.28 | 0.954 |
| Gluteal | 33 | 2.12 | 2.05 | 0.07 | 0.23 | 0.954 |
|  | 34 | 2.92 | 2.93 | -0.01 | 0.29 | 0.974 |
| Anterior Thigh | 35 | 1.85 | 1.82 | 0.03 | 0.27 | 0.974 |
|  | 36 | 1.11 | 1.06 | 0.05 | 0.29 | 0.917 |
|  | 37 | 0.75 | 0.75 | 0.00 | 0.09 | 0.983 |
|  | 38 | 1.43 | 1.46 | -0.02 | 0.14 | 0.973 |
|  | 39 | 1.17 | 1.17 | 0.00 | 0.11 | 0.977 |
|  | 40 | 1.22 | 1.21 | 0.01 | 0.13 | 0.968 |
|  | 41 | 1.88 | 1.90 | -0.02 | 0.27 | 0.933 |
|  | 42 | 1.80 | 1.77 | 0.03 | 0.21 | 0.940 |
|  | 43 | 1.62 | 1.67 | -0.05 | 0.47 | 0.810 |
| Posterior Thigh | 44 | 1.71 | 1.64 | 0.07 | 0.31 | 0.902 |
|  | 45 | 1.08 | 1.07 | 0.01 | 0.12 | 0.964 |
|  | 46 | 0.80 | 0.85 | -0.05 | 0.13 | 0.918 |
|  | 47 | 2.23 | 2.29 | -0.06 | 0.21 | 0.987 |
|  | 48 | 1.37 | 1.32 | 0.05 | 0.29 | 0.941 |
|  | 49 | 1.28 | 1.29 | -0.01 | 0.22 | 0.940 |
| Fossa poplitea | 50 | 0.67 | 0.68 | -0.01 | 0.12 | 0.900 |
| Anterior Lower Leg | 51 | 0.57 | 0.56 | 0.01 | 0.09 | 0.929 |
|  | 52 | 0.64 | 0.65 | -0.01 | 0.09 | 0.955 |
| Calf | 53 | 0.69 | 0.68 | 0.01 | 0.09 | 0.956 |
|  | 54 | 0.73 | 0.73 | 0.01 | 0.17 | 0.889 |
|  | 55 | 0.81 | 0.77 | 0.03 | 0.12 | 0.912 |
|  | 56 | 0.93 | 0.93 | -0.01 | 0.11 | 0.948 |
|  | SF_Total_ Depth | 66.70 | 66.57 | 0.13 | 1.57 | 0.998 |

SF_Total_ Depth: total depth of subcutaneous fat, S_D_: standard deviation of difference, d̅: mean of difference, ICC: Intraclass coefficient; C: caliper; US: ultrasound

Supplementary Table S5 Caliper’s Interrater Reliability (n=42)

| **Body part** | **Field**  **#** | **C1**  **(cm)** | **C2**  **(cm)** | **d̅**  **(cm)** | **s_D_**  **(cm)** | **ICC** |
| --- | --- | --- | --- | --- | --- | --- |
| Upper Arm | 1 | 0.65 | 0.56 | 0.08 | 0.20 | 0.804 |
|  | 2 | 0.43 | 0.48 | -0.05 | 0.15 | 0.861 |
|  | 3 | 0.39 | 0.38 | 0.01 | 0.14 | 0.808 |
|  | 4 | 0.75 | 0.98 | -0.23 | 0.24 | 0.709 |
|  | 5 | 1.05 | 0.89 | 0.16 | 0.27 | 0.774 |
|  | 6 | 0.73 | 0.60 | 0.12 | 0.15 | 0.884 |
| Lower Arm | 7 | 0.28 | 0.30 | -0.02 | 0.14 | 0.669 |
|  | 8 | 0.30 | 0.28 | 0.01 | 0.08 | 0.801 |
|  | 9 | 0.47 | 0.37 | 0.10 | 0.15 | 0.779 |
|  | 10 | 0.30 | 0.27 | 0.04 | 0.09 | 0.868 |
| Chest/Abdomen | 11 | 0.65 | 0.71 | -0.06 | 0.26 | 0.773 |
|  | 12* | 1.10 | 1.25 | -0.15 | 0.28 | 0.903 |
|  | 13 | 1.13 | 1.07 | 0.06 | 0.22 | 0.935 |
|  | 14 | 1.52 | 1.20 | 0.32 | 0.85 | 0.494 |
|  | 15 | 1.32 | 1.33 | -0.01 | 0.24 | 0.943 |
|  | 16 | 0.56 | 0.57 | -0.01 | 0.22 | 0.833 |
|  | 17* | 1.01 | 0.90 | 0.10 | 0.33 | 0.904 |
|  | 18 | 0.81 | 0.84 | -0.03 | 0.31 | 0.865 |
|  | 19 | 0.91 | 0.83 | 0.08 | 0.20 | 0.919 |
|  | 20 | 0.74 | 0.72 | 0.01 | 0.27 | 0.795 |
| Anterior Neck | 21 | 0.69 | 0.70 | -0.01 | 0.13 | 0.922 |
| Back | 22 | 0.25 | 0.29 | -0.04 | 0.07 | 0.741 |
|  | 23 | 0.99 | 1.00 | -0.02 | 0.13 | 0.973 |
|  | 24 | 0.75 | 0.74 | 0.01 | 0.12 | 0.958 |
|  | 25 | 0.94 | 0.87 | 0.07 | 0.15 | 0.947 |
|  | 26 | 0.99 | 0.90 | 0.09 | 0.15 | 0.934 |
|  | 27 | 1.17 | 1.12 | 0.05 | 0.29 | 0.869 |
|  | 28 | 0.79 | 0.87 | -0.08 | 0.20 | 0.807 |
|  | 29 | 0.91 | 0.90 | 0.01 | 0.25 | 0.881 |
|  | 30 | 0.81 | 0.83 | -0.01 | 0.10 | 0.973 |
|  | 31 | 0.79 | 0.80 | -0.01 | 0.21 | 0.883 |
| Posterior Neck | 32 | 1.35 | 1.33 | 0.02 | 0.50 | 0.527 |
| Gluteal | 33 | 1.58 | 1.62 | -0.04 | 0.44 | 0.699 |
|  | 34 | 1.37 | 1.54 | -0.17 | 0.43 | 0.774 |
| Anterior Thigh | 35 | 1.28 | 1.14 | 0.14 | 0.34 | 0.845 |
|  | 36 | 0.98 | 0.86 | 0.11 | 0.23 | 0.914 |
|  | 37 | 0.66 | 0.61 | 0.06 | 0.28 | 0.774 |
|  | 38 | 1.14 | 1.01 | 0.14 | 0.28 | 0.803 |
|  | 39 | 1.10 | 0.95 | 0.15 | 0.24 | 0.859 |
|  | 40 | 1.04 | 0.89 | 0.15 | 0.22 | 0.853 |
|  | 41 | 0.87 | 1.01 | -0.15 | 0.29 | 0.701 |
|  | 42 | 1.24 | 1.27 | -0.03 | 0.21 | 0.906 |
|  | 43 | 1.40 | 1.24 | 0.16 | 0.28 | 0.841 |
| Posterior Thigh | 44 | 1.30 | 1.21 | 0.09 | 0.24 | 0.846 |
|  | 45 | 0.98 | 0.78 | 0.20 | 0.21 | 0.776 |
|  | 46 | 0.78 | 0.62 | 0.16 | 0.24 | 0.558 |
|  | 47 | 1.53 | 1.32 | 0.21 | 0.48 | 0.727 |
|  | 48 | 1.26 | 0.94 | 0.33 | 1.25 | 0.184 |
|  | 49 | 0.79 | 0.77 | 0.02 | 0.22 | 0.869 |
| Fossa poplitea | 50 | 0.68 | 0.44 | 0.24 | 0.26 | 0.300 |
| Anterior Lower Leg | 51 | 0.62 | 0.48 | 0.14 | 0.15 | 0.697 |
|  | 52 | 0.56 | 0.50 | 0.06 | 0.12 | 0.902 |
| Calf | 53 | 0.73 | 0.57 | 0.16 | 0.15 | 0.769 |
|  | 54 | 0.79 | 0.54 | 0.25 | 0.20 | 0.652 |
|  | 55 | 0.77 | 0.61 | 0.16 | 0.16 | 0.737 |
|  | 56 | 0.88 | 0.76 | 0.12 | 0.19 | 0.813 |
|  | SF_Total_ Depth | 48.92 | 45.62 | 3.31 | 3.12 | 0.966 |

SF_Total_ Depth: total depth of subcutaneous fat, S_D_: standard deviation of difference, d̅: mean of difference, ICC: Intraclass coefficient; C: caliper, *only male subjects n=24

Supplementary Table S6 US Interrater reliability (n=44)

| **Body part** | **Field**  **#** | **US 1 (cm)** | **US 2 (cm)** | **d̅**  **(cm)** | **s_D_**  **(cm)** | **ICC** |
| --- | --- | --- | --- | --- | --- | --- |
| Upper Arm | 1 | 0.53 | 0.62 | -0.09 | 0.13 | 0.855 |
|  | 2 | 0.79 | 0.87 | -0.09 | 0.23 | 0.889 |
|  | 3 | 0.55 | 0.56 | -0.01 | 0.14 | 0.880 |
|  | 4 | 0.70 | 1.18 | -0.48 | 0.35 | 0.378 |
|  | 5 | 1.20 | 1.16 | 0.04 | 0.23 | 0.917 |
|  | 6 | 1.07 | 0.94 | 0.13 | 0.27 | 0.849 |
| Lower Arm | 7 | 0.31 | 0.44 | -0.13 | 0.19 | 0.484 |
|  | 8 | 0.34 | 0.43 | -0.09 | 0.14 | 0.746 |
|  | 9 | 0.59 | 0.73 | -0.15 | 0.19 | 0.780 |
|  | 10 | 0.35 | 0.42 | -0.08 | 0.21 | 0.667 |
| Chest/Abdomen | 11 | 1.21 | 1.37 | -0.16 | 0.49 | 0.794 |
|  | 12 | 1.92 | 2.26 | -0.34 | 0.53 | 0.888 |
|  | 13 | 1.35 | 1.53 | -0.19 | 0.30 | 0.927 |
|  | 14 | 2.11 | 2.10 | 0.01 | 0.30 | 0.971 |
|  | 15 | 2.67 | 2.78 | -0.10 | 0.44 | 0.945 |
|  | 16 | 1.00 | 1.11 | -0.11 | 0.37 | 0.774 |
|  | 17 | 2.36 | 2.59 | -0.23 | 0.78 | 0.886 |
|  | 18 | 1.18 | 1.22 | -0.04 | 0.30 | 0.931 |
|  | 19 | 1.39 | 1.22 | 0.17 | 0.29 | 0.897 |
|  | 20 | 1.36 | 1.27 | 0.10 | 0.67 | 0.754 |
| Anterior Neck | 21 | 0.58 | 0.69 | -0.11 | 0.17 | 0.781 |
| Back | 22 | 0.40 | 0.48 | -0.08 | 0.18 | 0.618 |
|  | 23 | 0.90 | 0.92 | -0.02 | 0.27 | 0.831 |
|  | 24 | 0.79 | 0.79 | 0.00 | 0.20 | 0.877 |
|  | 25 | 0.98 | 0.99 | -0.01 | 0.29 | 0.854 |
|  | 26 | 1.06 | 1.12 | -0.06 | 0.25 | 0.897 |
|  | 27 | 1.66 | 1.44 | 0.22 | 0.58 | 0.662 |
|  | 28 | 1.00 | 1.03 | -0.04 | 0.34 | 0.722 |
|  | 29 | 1.08 | 1.07 | 0.01 | 0.27 | 0.895 |
|  | 30 | 1.03 | 1.04 | -0.01 | 0.30 | 0.843 |
|  | 31 | 0.92 | 0.96 | -0.03 | 0.29 | 0.848 |
| Posterior Neck | 32 | 2.64 | 2.30 | 0.34 | 1.02 | 0.593 |
| Gluteal | 33 | 1.97 | 2.03 | -0.05 | 0.45 | 0.818 |
|  | 34 | 2.35 | 2.49 | -0.14 | 0.68 | 0.829 |
| Anterior Thigh | 35 | 1.48 | 1.54 | -0.06 | 0.66 | 0.767 |
|  | 36 | 1.10 | 1.08 | 0.02 | 0.37 | 0.894 |
|  | 37 | 0.79 | 0.74 | 0.05 | 0.12 | 0.970 |
|  | 38 | 1.59 | 1.42 | 0.17 | 0.34 | 0.820 |
|  | 39 | 1.26 | 1.16 | 0.10 | 0.17 | 0.930 |
|  | 40 | 1.27 | 1.20 | 0.08 | 0.18 | 0.937 |
|  | 41 | 1.66 | 1.89 | -0.23 | 0.53 | 0.735 |
|  | 42 | 1.71 | 1.80 | -0.09 | 0.05 | 0.868 |
|  | 43 | 1.89 | 1.57 | 0.32 | 0.55 | 0.649 |
| Posterior Thigh | 44 | 1.71 | 1.60 | 0.11 | 0.24 | 0.914 |
|  | 45 | 1.08 | 1.04 | 0.04 | 0.20 | 0.902 |
|  | 46 | 0.78 | 0.79 | -0.01 | 0.17 | 0.888 |
|  | 47 | 2.41 | 2.03 | 0.38 | 0.34 | 0.916 |
|  | 48 | 1.40 | 1.26 | 0.14 | 0.26 | 0.935 |
|  | 49 | 1.14 | 1.19 | -0.05 | 0.35 | 0.833 |
| Fossa poplitea | 50 | 0.71 | 0.67 | 0.03 | 0.21 | 0.744 |
| Anterior Lower Leg | 51 | 0.63 | 0.54 | 0.09 | 0.15 | 0.786 |
|  | 52 | 0.66 | 0.60 | 0.06 | 0.13 | 0.890 |
| Calf | 53 | 0.75 | 0.64 | 0.11 | 0.12 | 0.881 |
|  | 54 | 0.77 | 0.68 | 0.09 | 0.12 | 0.912 |
|  | 55 | 0.81 | 0.80 | 0.01 | 0.15 | 0.896 |
|  | 56 | 0.92 | 0.91 | 0.01 | 0.19 | 0.862 |
|  | **SF_Total_ Depth** | 66.81 | 67.25 | -0.45 | 4.82 | 0.983 |

SF_Total_ Depth: total depth of subcutaneous fat, S_D_: standard deviation of difference, d̅: mean of difference, ICC: Intraclass coefficient; US: ultrasound

Supplementary Table S7 Caliper vs. US (n=53)

| **Body part** | **Field**  **#** | **C**  **(cm)** | **US (cm)** | **d̅**  **(cm)** | **d̅**  **(%)** | **s_D_**  **(cm)** | **ICC** |
| --- | --- | --- | --- | --- | --- | --- | --- |
| Upper Arm | 1 | 0.54 | 0.59 | -0.05 | -8.00 | 0.11 | 0.903 |
|  | 2 | 0.47 | 0.83 | -0.36 | -43.68 | 0.32 | 0.504 |
|  | 3 | 0.37 | 0.54 | -0.17 | -31.03 | 0.16 | 0.606 |
|  | 4 | 0.96 | 1.13 | -0.18 | -15.45 | 0.29 | 0.740 |
|  | 5 | 0.91 | 1.11 | -0.21 | -18.51 | 0.33 | 0.662 |
|  | 6 | 0.61 | 0.89 | -0.28 | -31.56 | 0.24 | 0.645 |
| Lower Arm | 7 | 0.29 | 0.40 | -0.11 | -27.54 | 0.14 | 0.654 |
|  | 8 | 0.28 | 0.39 | -0.11 | -28.82 | 0.11 | 0.670 |
|  | 9 | 0.37 | 0.68 | -0.31 | -45.83 | 0.20 | 0.444 |
|  | 10 | 0.26 | 0.38 | -0.12 | -32.35 | 0.16 | 0.604 |
| Chest/Abdomen | 11 | 0.69 | 1.27 | -0.57 | -45.24 | 0.44 | 0.500 |
|  | 12* | 1.19 | 1.27 | -0.08 | -6.36 | 0.35 | 0.628 |
|  | 13 | 0.97 | 1.35 | -0.38 | -28.40 | 0.49 | 0.693 |
|  | 14 | 1.10 | 1.85 | -0.75 | -40.70 | 0.70 | 0.542 |
|  | 15 | 1.20 | 2.49 | -1.29 | -51.98 | 0.76 | 0.416 |
|  | 16 | 0.53 | 1.04 | -0.52 | -49.40 | 0.42 | 0.416 |
|  | 17* | 0.84 | 1.50 | -0.66 | -43.96 | 0.69 | 0.573 |
|  | 18 | 0.74 | 1.06 | -0.32 | -29.97 | 0.41 | 0.715 |
|  | 19 | 0.77 | 1.10 | -0.33 | -30.24 | 0.33 | 0.700 |
|  | 20 | 0.68 | 1.14 | -0.47 | -41.01 | 0.49 | 0.414 |
| Anterior Neck | 21 | 0.65 | 0.64 | 0.02 | 2.88 | 0.12 | 0.912 |
| Back | 22 | 0.27 | 0.45 | -0.17 | -38.61 | 0.16 | 0.406 |
|  | 23 | 0.93 | 0.84 | 0.09 | 10.50 | 0.28 | 0.827 |
|  | 24 | 0.69 | 0.72 | -0.03 | -4.21 | 0.16 | 0.903 |
|  | 25 | 0.80 | 0.90 | -0.11 | -11.89 | 0.27 | 0.825 |
|  | 26 | 0.85 | 1.01 | -0.17 | -16.53 | 0.19 | 0.869 |
|  | 27 | 1.07 | 1.34 | -0.27 | -20.41 | 0.35 | 0.716 |
|  | 28 | 0.83 | 0.99 | -0.16 | -16.17 | 0.28 | 0.696 |
|  | 29 | 0.84 | 0.97 | -0.13 | -13.36 | 0.28 | 0.838 |
|  | 30 | 0.76 | 0.94 | -0.18 | -19.42 | 0.22 | 0.796 |
|  | 31 | 0.75 | 0.86 | -0.11 | -13.28 | 0.29 | 0.773 |
| Posterior Neck | 32 | 1.34 | 2.31 | -0.97 | -41.87 | 1.00 | 0.204 |
| Gluteal | 33 | 1.71 | 2.10 | -0.39 | -18.55 | 0.46 | 0.642 |
|  | 34 | 1.62 | 2.87 | -1.26 | -43.78 | 0.68 | 0.411 |
| Anterior Thigh | 35 | 1.27 | 1.76 | -0.49 | -27.91 | 0.52 | 0.695 |
|  | 36 | 0.98 | 1.06 | -0.08 | -7.06 | 0.40 | 0.802 |
|  | 37 | 0.67 | 0.71 | -0.04 | -5.29 | 0.16 | 0.918 |
|  | 38 | 1.05 | 1.40 | -0.35 | -24.89 | 0.33 | 0.631 |
|  | 39 | 1.04 | 1.13 | -0.10 | -8.44 | 0.30 | 0.800 |
|  | 40 | 0.96 | 1.19 | -0.24 | -19.80 | 0.21 | 0.793 |
|  | 41 | 1.01 | 1.85 | -0.83 | -45.04 | 0.52 | 0.297 |
|  | 42 | 1.28 | 1.79 | -0.51 | -28.46 | 0.36 | 0.537 |
|  | 43 | 1.28 | 1.60 | -0.32 | -20.05 | 0.45 | 0.676 |
| Posterior Thigh | 44 | 1.31 | 1.67 | -0.37 | -21.82 | 0.34 | 0.73 |
|  | 45 | 0.87 | 1.05 | -0.19 | -17.67 | 0.25 | 0.728 |
|  | 46 | 0.65 | 0.77 | -0.12 | -15.93 | 0.24 | 0.588 |
|  | 47 | 1.44 | 2.16 | -0.73 | -33.55 | 0.73 | 0.603 |
|  | 48 | 1.04 | 1.32 | -0.29 | -21.62 | 0.37 | 0.795 |
|  | 49 | 0.86 | 1.24 | -0.38 | -30.70 | 0.82 | 0.645 |
| Fossa poplitea | 50 | 0.46 | 0.65 | -0.20 | -30.29 | 0.18 | 0.564 |
| Anterior Lower Leg | 51 | 0.51 | 0.56 | -0.05 | -8.69 | 0.13 | 0.831 |
|  | 52 | 0.54 | 0.63 | -0.09 | -14.73 | 0.11 | 0.872 |
| Calf | 53 | 0.62 | 0.68 | -0.07 | -9.72 | 0.17 | 0.835 |
|  | 54 | 0.58 | 0.73 | -0.15 | -20.70 | 0.19 | 0.745 |
|  | 55 | 0.62 | 0.80 | -0.17 | -21.52 | 0.14 | 0.737 |
|  | 56 | 0.79 | 0.93 | -0.14 | -14.92 | 0.18 | 0.781 |
|  | SF_Total_ Depth | 45.60 | 62.21 | -16.61 | -26.86 | 8.31 | 0.581 |

SF_Total_ Depth: total depth of subcutaneous fat, S_D_: standard deviation of difference, d̅: mean of difference, ICC: Intraclass coefficient; C: caliper; US: ultrasound, *only male subjects n=24
